# Transcriptional Waves in the Yeast Cell Cycle

**DOI:** 10.1371/journal.pbio.0030243

**Published:** 2005-06-28

Mobilizing an army to march into battle requires the increased activity of hundreds of people, from the quartermaster to the gunnery captain. The stately march of the cell cycle—from the first growth phase, through DNA synthesis, to the second growth phase, and on to mitosis and cell division—also demands increased activity, but of hundreds of genes, from histones to protein kinases. And just as the army must coordinate the shipment of C rations with the movement of its troops, so must the cell coordinate its genetic activities to ensure that raw materials and regulatory molecules are present where and when they are needed. In this issue, Janet Leatherwood, Bruce Futcher, and colleagues describe the waves of gene activity that accompany the phases of the cell cycle in the yeast Schizosaccharomyces pombe.

Using microarrays, the authors examined the expression level of 5,000 genes over the course of the cell cycle. They found that well over 2,000 of these genes undergo slight but observable and statistically meaningful oscillations. Of these, they chose to examine the top 750, an admittedly arbitrary cutoff that nonetheless highlights those whose expression levels rise and fall the most. They identified two broad waves of oscillation, one peaking in early to mid-G2 (the second growth phase) and the other late in G2 at the transition to mitosis. These two peaks were seen even in the 4,000 least cyclic genes, suggesting that many genes may be slightly upregulated not for adaptive purposes, but simply because some transcription factors inevitably go astray whenever there are lots of them around.

Such broad waves of upregulation are likely due to a simultaneous increase in the activity of multiple clusters of genes, each controlled by separate groups of transcription factors. A variety of cell culture manipulations allowed the researchers to identify eight clusters of genes, the activity of whose members was tightly co-regulated. (In this case, “cluster” refers not to genes physically grouped together on a chromosome, but to genes that are regulated similarly.) Scouring the promoters of these genes confirmed that each cluster was characterized by unique transcription factor binding sites. They also discovered that, as a group, these promoters tended to be longer than average, suggesting they may be more complex than those in non-oscillating genes.

The number of genes within each cluster ranged from only a few to over 100. The largest of them, the Cdc15 cluster, contains genes involved in mitosis, cytokinesis, and formation of the septum that separates the daughter cells, as well as genes for other functions. Other clusters regulate DNA replication, cell separation, synthesis of the histone proteins that act as spools on which DNA is wound, protein folding and stress response, ribosome biogenesis, and other aspects of the cell cycle.

Two other recent studies in S. pombe have found broadly similar patterns, and have identified 407 and 747 genes, respectively, as strong oscillators. There was a heartening degree of overlap, with 171 genes identified by all three studies, and 360 more found in two of the three. Even genes that made the cut in only one study were found likely to oscillate in the other two. Follow-up studies to further explore genes that are coordinated during the cell cycle march should help us to understand how the army of molecules exerts such fine control over both normal and abnormal cell growth and proliferation.

